# Distinct epigenomic and transcriptomic modifications associated with *Wolbachia*-mediated asexuality

**DOI:** 10.1371/journal.ppat.1008397

**Published:** 2020-03-18

**Authors:** Xin Wu, Amelia R. I. Lindsey, Paramita Chatterjee, John H. Werren, Richard Stouthamer, Soojin V. Yi

**Affiliations:** 1 School of Biological Sciences, Institute for Bioengineering and Bioscience, Georgia Institute of Technology, Atlanta, Georgia, United States of America; 2 Department of Entomology, University of California Riverside, Riverside, California, United States of America; 3 Current Address: Department of Biology, Indiana University, Bloomington, Indiana, United States of America; 4 Department of Biology, University of Rochester, Rochester, New York, United States of America; Pennsylvania State University, UNITED STATES

## Abstract

*Wolbachia* are maternally transmitted intracellular bacteria that induce a range of pathogenic and fitness-altering effects on insect and nematode hosts. In parasitoid wasps of the genus *Trichogramma*, *Wolbachia* infection induces asexual production of females, thus increasing transmission of *Wolbachia*. It has been hypothesized that *Wolbachia* infection accompanies a modification of the host epigenome. However, to date, data on genome-wide epigenomic changes associated with *Wolbachia* are limited, and are often confounded by background genetic differences. Here, we took sexually reproducing *Trichogramma* free of *Wolbachia* and introgressed their genome into a *Wolbachia*-infected cytoplasm, converting them to *Wolbachia*-mediated asexuality. *Wolbachia* was then cured from replicates of these introgressed lines, allowing us to examine the genome-wide effects of wasps newly converted to asexual reproduction while controlling for genetic background. We thus identified gene expression and DNA methylation changes associated with *Wolbachia*-infection. We found no overlaps between differentially expressed genes and differentially methylated genes, indicating that *Wolbachia*-infection associated DNA methylation change does not directly modulate levels of gene expression. Furthermore, genes affected by these mechanisms exhibit distinct evolutionary histories. Genes differentially methylated due to the infection tended to be evolutionarily conserved. In contrast, differentially expressed genes were significantly more likely to be unique to the *Trichogramma* lineage, suggesting host-specific transcriptomic responses to infection. Nevertheless, we identified several novel aspects of *Wolbachia*-associated DNA methylation changes. Differentially methylated genes included those involved in oocyte development and chromosome segregation. Interestingly, *Wolbachia*-infection was associated with higher levels of DNA methylation. Additionally, *Wolbachia* infection reduced overall variability in gene expression, even after accounting for the effect of DNA methylation. We also identified specific cases where alternative exon usage was associated with DNA methylation changes due to *Wolbachia* infection. These results begin to reveal distinct genes and molecular pathways subject to *Wolbachia* induced epigenetic modification and/or host responses to *Wolbachia*-infection.

## Author summary

*Wolbachia* is an extremely common endosymbiotic infection of arthropods and nematodes. One of the reasons why *Wolbachia* can so successfully infect diverse species is the bacterium’s ability to profoundly alter the reproductive behavior of its host. It has been proposed that *Wolbachia* may modify host’s epigenetic programs to alter its reproductive behavior. However, it has been difficult to study how epigenetic programs change with *Wolbachia* infection, due to the confounding effects of genetic backgrounds. Here, we studied host transcriptome and epigenome changes associated with *Wolbachia* infection in a homogenous genetic background, by carrying out an innovative introgression scheme. By doing so, we show, for the first time, high-resolution molecular consequences of intracellular infection and offer insights into epigenetic and transcriptomic regulation of invertebrates.

## Introduction

*Wolbachia* is a widespread endosymbiont of arthropods and nematodes, estimated to infect 40 to 60 percent of all insect species [1, 2]. Its success can be attributed to wide ranging effects on host fitness, including reproductive parasitism [3]. For example, in parasitoid wasps of the genus *Trichogramma*, *Wolbachia* converts wasp hosts to an asexual mode of reproduction [4–6]. In uninfected wasps, males develop from haploid, unfertilized eggs, and females only result from eggs that were converted to diploidy via fertilization. When the wasps are infected with *Wolbachia*, haploid unfertilized eggs are converted to diploidy through a fertilization-independent mechanism, resulting in the asexual production of female offspring. This shift in reproductive mode enables the spread of *Wolbachia* throughout a population [7]. In some cases, *Trichogramma* wasps become dependent upon *Wolbachia* for the production of female offspring. In this scenario, wasps lose the ability to fertilize their eggs, and without *Wolbachia*-mediated diploidization, no females are produced [7]. This phenomenon is increasingly referred to as “symbiont addiction”: the infection by the symbiont leads to an evolutionary response that creates a dependency on *Wolbachia* where no such need existed prior to the onset of the symbiotic relationship [8, 9].

While initial discoveries of *Wolbachia*’s ubiquity and control of host reproduction were made more than two decades ago, molecular details of how *Wolbachia* manipulates its host to maintain a persistent infection and alter host reproduction still remain relatively poorly understood, and especially so for *Wolbachia* that induce parthenogenesis. Genes associated with *Wolbachia’s* prophage are responsible for inducing cytoplasmic incompatibility [10–12], and potentially male-killing [13]. However, the *Wolbachia* strains infecting *Trichogramma* have no prophage [14, 15], do not contain orthologs of these known reproductive manipulator loci [12], and only have remnants of other prophage genes [15]. While the loci behind parthenogenesis induction have not been described, it is known that in *Trichogramma* wasps, *Wolbachia* prevents chromosome segregation during the first mitotic division of unfertilized eggs to create diploid offspring [16].

One of the mechanisms that may have relevance to *Wolbachia*’s manipulation of the host is epigenetic modification. It has been long thought that *Wolbachia* may modify host “epigenomes”: heritable chemical modifications of DNA and histones affecting chromatin conformation [17]. For example, in *Drosophila simulans*, *Wolbachia* infection is known to affect the chromatin reorganization during spermatogenesis [18]. In a leafhopper species, *Wolbachia* infection can interfere with the host genome’s imprinting pattern [19]. More recently, *Wolbachia* infection was shown to alter the expression of host methylation machinery in *Aedes aegypti* [20, 21], *Drosophila melanogaster* [22] and *Cotesia plutellae* [23]. These studies indicate that *Wolbachia* infection might be accompanied by a systematic modification of the host epigenome, including changes in DNA methylation. However, how *Wolbachia* affect the epigenome (such as genomic DNA methylation) at fine-scale has been difficult to resolve, due to several problems. First, model insects such as *Drosophila* and other Diptera (e.g., mosquitoes) harbor little genomic DNA methylation [24]. Second, epigenetic patterns are strongly influenced by genetic background [25, 26], thus it is not straightforward to disentangle the effects of *Wolbachia* infection and genetic background.

The *Trichogramma pretiosum* genome harbors a full set of DNA methylation machinery and exhibits genomic cytosine methylation [27], thus making it an excellent system to investigate how the host methylome changes with *Wolbachia* infection. *Trichogramma* wasps are geographically widespread, and genetically variable. Consequently, comparative studies of different *Trichogramma* methylomes would be confounded by genomic differences and each line’s adaptation to its environment. Furthermore, the dependence of fully parthenogenetic *Trichogramma* on their resident *Wolbachia* precludes antibiotic curing to create comparable *Wolbachia*-free lines. In some lines of *Trichogramma*, there is a complete loss of fertilization. Here, we use a *Wolbachia*-infected *Trichogramma* line with a significantly impaired rate of fertilization: females do not fertilize enough eggs to maintain a self-sustaining population in the absence of *Wolbachia* [28]. However, a low level of egg fertilization allows us to introgress genetic material from a sexual population by back-crossing, eventually replacing the mutations that are responsible for the impaired fertilization rate. The result of this introgression is a line of wasps newly converted to *Wolbachia*-mediated asexuality, which can also be converted back to sexual reproduction through antibiotic treatment. This scheme allowed us to profile the molecular signatures of *Wolbachia* infection in *Trichogramma* hosts in a genetically homogeneous setting. Here, for the first time, we show detailed genome-wide transcriptome and methylome modifications associated with *Wolbachia* infection.

## Results

### Introgression of a sexual nuclear genome into *Wolbachia*-infected asexual cytoplasm

**Fig 1:**
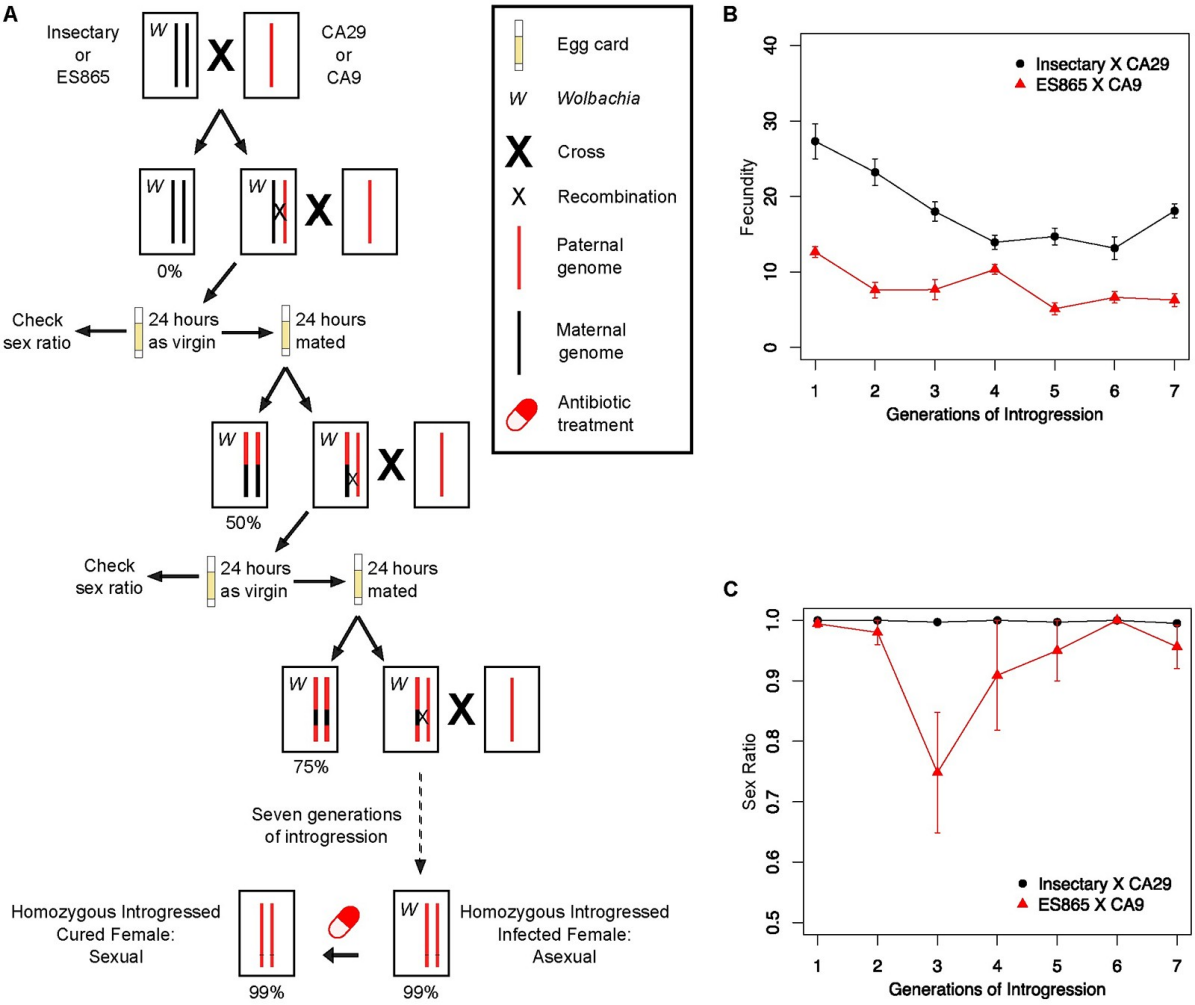
Crossing scheme for creating homozygous introgressed isofemale lines and fitness at each introgression generation. A) After seven generations of introgression, between 95–99% of the *Wolbachia*-dependent asexual nuclear background was replaced by the sexually reproducing nuclear background. At each generation of the introgression, we used virgin wasps to assay for sex ratio (a proxy for successful *Wolbachia*-mediated parthenogenesis) and fecundity prior to mating with a sexual male. All offspring were screened for zygosity, allowing for the identification of females produced via fertilization rather than *Wolbachia*-mediated parthenogenesis. This scheme was performed 3 independent times to generate 3 isofemale lines. B) Wasp fitness and C) efficiency of *Wolbachia*-mediated parthenogenesis at each generation of introgression. Sex ratio is denoted as the proportion of female offspring.

For these experiments, we used four isofemale lines of *Trichogramma pretiosum*: two *Wolbachia*-infected asexual lines (termed “Insectary” and ES865”), and two *Wolbachia*-free sexually reproducing lines (“CA29” and “CA9”). We introgressed the genome derived from a *Wolbachia*-free, sexually reproducing *Trichogramma* line into a cytoplasm derived from a *Wolbachia* infected, asexually reproducing *Trichogramma* (CA29 genome into Insectary cytoplasm, and CA9 into ES865) (Fig 1A). Introgression pairs (Insectary X CA29, and ES865 X CA9) were chosen based on the ability to track introgression using a molecular marker (Methods). The fecundity of wasps significantly decreased over each generation of the introgression protocol, consistent with the expectation of cyto-nuclear incompatibilities (Fig 1B; GLM: Insectary: χ^2^ = 33.701, *P* < 0.0001; ES865: χ^2^ = 44.372, *P* < 0.0001). Across the duration of the introgression, there was no significant change in the sex ratio of offspring produced by *Wolbachia*-infected virgin females, indicating successful induction of *Wolbachia*-mediated parthenogenesis in the new backgrounds (Fig 1C; GLM: Insectary: χ^2^ = 1.527, *P* = 0.2166; ES865: χ^2^ = 2.943, *P* = 0.0862). The ES865 X CA9 introgressed wasps were less fecund than the Insectary X CA29 introgressed wasps, and we were unable to expand the colonies sufficiently for further experimentation. This is consistent with the previously observed disadvantageous cyto-nuclear interactions commonly found in some *Trichogramma* crosses [4–6, 16]. Thus, we chose the Insectary X CA29 introgressed lines for transcriptomic and epigenetic analysis. Three independently generated Insectary X CA29 introgression lines were maintained, and individual replicates of each introgressed line were cured of their *Wolbachia* infections using antibiotics, restoring sexual reproduction. Following curing, virgin females produced only male offspring in the cured introgressed lines, whereas mated females produced both sons and daughters. There is no evidence for any other microbes in these lines of lab-reared *Trichogramma* [15, 27], so only *Wolbachia* infection status differs between the cured and infected replicates. *Wolbachia* infection status in all lines was additionally confirmed with PCR. DNA and RNA were extracted from *Wolbachia*-infected and cured introgressed lines for whole genome bisulfite sequencing (WGBS) and RNA-Seq (Methods).

We utilized the genome sequences of the parental lines (Insectary and CA29) and the WGBS data of the introgressed lines to explicitly map the genomic profiles of introgression. Specifically, using a tool that can identify single nucleotide polymorphism at the genomic level from WGBS data [29], we examined the origin of SNPs in each introgressed line and determined whether they originated from the paternal (introgressed) or maternal (non-introgressed) genomes, and estimated the total percentage of non-introgressed regions in each line (Methods). Based on this approach, two of the three lines (“B” and “C”) showed > 99% introgression (S1 Table). In the third line (line “A”), approximately 5–8% of the genome was of maternal origin, retained from the original asexual maternal line (S1 Table). The reason for the lower level introgression in this line may be that our introgression scheme required tracking a maternal marker through the course of the introgression. This would result in linked material regions being maintained in the introgressed lines. In establishing the final lines, progeny were chosen randomly (rather than again screening for parentage at the A9 locus), as we no longer needed to track fertilization after that point. For controlled comparisons, these non-introgressed regions were excluded from all lines for further transcriptome and methylome analyses. However, including or excluding regions that harbored putatively non-introgressed regions led to similar results (S2 Table), and it should be noted that the genomic make-up is identical between infected and cured versions of the same line.

### DNA methylation modification of *T*. *pretiosum* associated with *Wolbachia*

**Fig 2:**
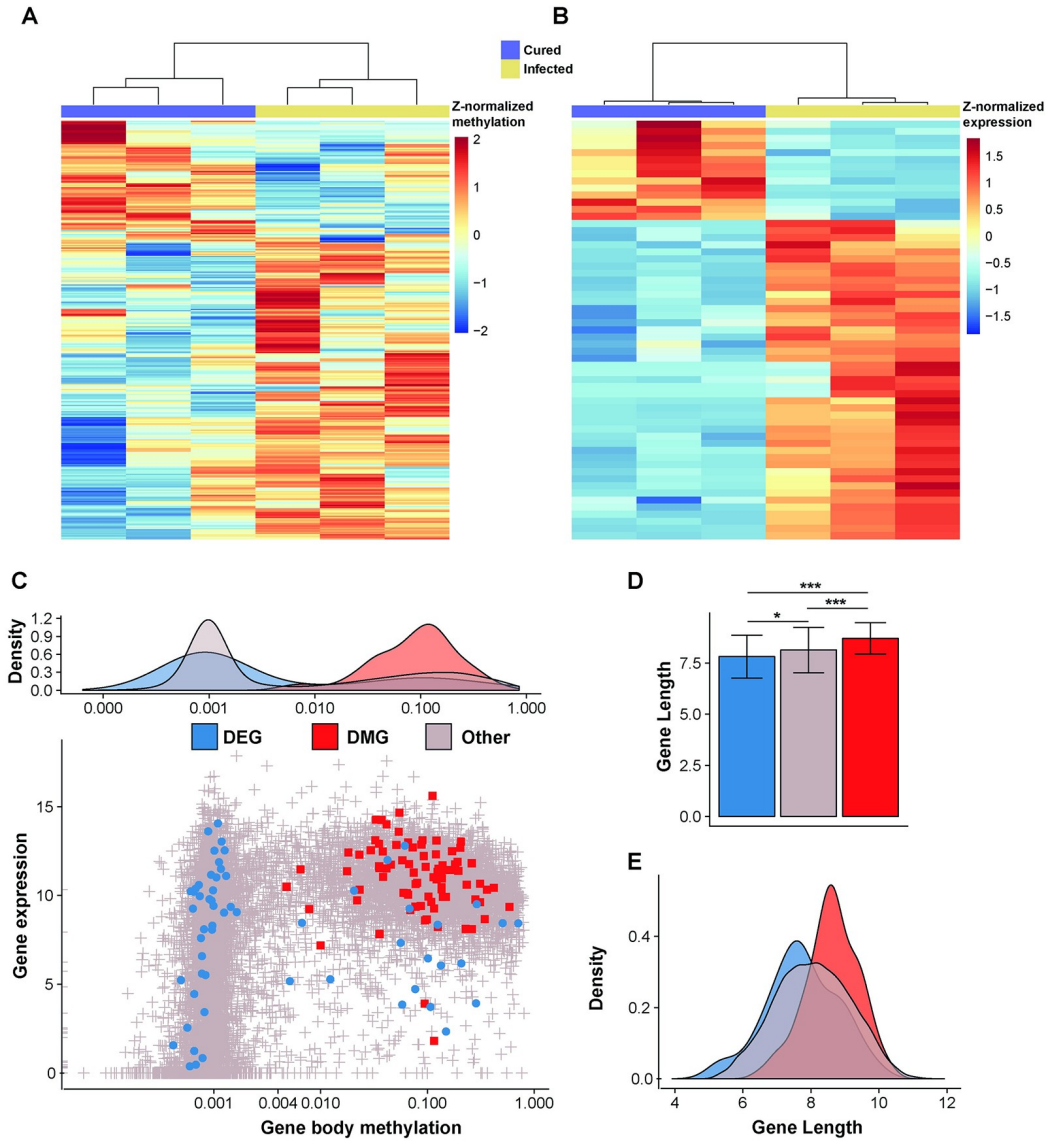
Differential methylation and expression between *Wolbachia* infected and cured lines. A) Heatmap of 340 significantly differentially methylated positions (DMPs, found within 84 genes) detected using RADMeth. More sites exhibit increased DNA methylation in the infected lines (239 out of 340 DMPs are hypermethylated in the infected lines compared to cured lines). B) Heatmap of 59 differentially expressed genes, the majority (39 out of 59; χ^2^, *P* = 3.13x10^-6^) of which were up-regulated in the infected lines. C) Gene body fractional methylation and gene expression relationship between DEG, DMG and other genes (genes that are neither DEG or DMG) accompanied by gene body fractional methylation densities of each gene class. Gene body fractional methylation is calculated as the mean fractional methylation of all CpGs within each gene, and shows a bimodal distribution as in other species [46]. Gene body fractional methylation and gene expression are log_10_ and log_2_ transformed, respectively, to improve normality. D) Average gene lengths and E) density plot of gene lengths for each gene class. Error bars represent standard deviations.

We first assessed genome-wide cytosine methylation profiles of the introgressed lines with and without *Wolbachia*. There were 106,475 cytosines that were methylated in at least one of the six samples (originating from the three cured and three infected lines), identified using the software Bis-Class [30] and all of which had exceptional sequencing depth (S3 Table). We first identified individual cytosine positions that were significantly differentially methylated, referred to as “differentially methylated positions” (**DMPs**), between the two lines (Methods, S4 Table). We identified a total of 340 DMPs (FDR-adjusted *Q* < 0.05), 317 of which were genic and 23 of which were intergenic. Interestingly, we found that 238 (70%) of DMPs had significantly higher levels of fractional methylation (the frequency of methylated reads out of total reads for a specific cytosine) in the infected lines compared to the cured lines (χ^2^ test, *P* < 10^−15^; Fig 2A). The 84 genes harboring 317 genic DMPs were then defined as “differentially methylated genes” (**DMGs**), whereas the remaining 23 intergenic DMPs were characterized by the nearest annotated gene (S4 Table).

DMGs contained, on average, 4.5 DMPs (S4 Table). The gene containing the most DMPs had 18 (S1 Fig) despite it not being a particularly long gene (7,439 bp or 74^th^ percentile of all gene lengths; Fig 2D and 2E). This gene, TPRE002596 (homolog of *Drosophila* CG4896), is implicated in alternative mRNA splicing in *Drosophila melanogaster* [31]. DMGs also included three genes known to be involved in polarity formation in early development, namely TPRE005355 (homolog of *D*. *melanogaster Rab6*, containing 4 DMPs,) TPRE001344 (homolog of *D*. *melanogaster CG7483*, containing 10 DMPs), and TPRE000491, harboring 2 DMPs. *Rab6* is critical in *D*. *melanogaster* actin organization and polarization of the oocyte cytoskeleton [32], and CG7483 is predicted to influence *oskar* mRNA localization at the posterior pole of oocytes in *D*. *melanogaster* [33]. TPRE000491 contains a Toll/interleukin-1 receptor domain, which is involved in dorso-ventral polarity formation in *D*. *melanogaster* embryos [34, 35].

Largely due to these three genes, DMGs showed significant gene ontology enrichment for terms relating to embryonic axis specification, embryonic pattern specification, and oocyte anterior/posterior axis specification (S5 Table). Additionally, several other genes containing high numbers of DMPs also had functions relating to embryonic development, despite not being annotated with those specific ontology terms (S6 Table). One of these genes, TPRE012191 (homolog of *D*. *melanogaster Chc*), has the second most DMPs of any gene (n = 13) and plays a key role in yolk protein uptake in the developing oocyte [36]. These results align with previous studies suggesting epigenetic modification by *Wolbachia* [20–22, 37], as well as the proposed functional association of *Wolbachia* with the germline to facilitate transmission [37–39].

### *Wolbachia* infection results in changes in gene expression and exon usage

Next, we assessed the transcriptomic signatures of *Wolbachia* infection in the introgressed *Trichogramma* lines. Using a negative binomial generalized linear model method [40], we identified 59 genes that were significantly differentially expressed (DEGs) between *Wolbachia*-infected and cured lines (FDR-adjusted *Q* < 0.05)(Fig 2B; S2 Fig). Of these, 45 (76%) genes were up-regulated in the infected lines (χ^2^ test, *P* < 10^−15^), with an average of 4.72-fold change higher expression relative to the cured lines (S4 Table). Genes that were down-regulated in the *Wolbachia*-infected lines (24%) had an average of 3.47-fold lower expression relative to the cured lines. Interestingly, differentially expressed genes were not significantly overrepresented for any gene ontology terms, partially because the majority of the DEGs had no ontology terms assigned to them at all. Indeed, 35 of the 59 DEGs had previously been classified as lineage-specific in *Trichogramma* [27], which is a significant enrichment (Fisher’s exact test, *P* = 1.29X10^-7^) (S7 Table). The overrepresentation of DEGs with unique evolutionary patterns in the *Trichogramma* lineage points towards either a host-specific method of manipulation by *Wolbachia*, or a host response to *Wolbachia* infection that is unique to *Trichogramma*.

Next, we examined patterns of differential exon usage associated with *Wolbachia* infection status, using a method that models exon usage fold changes based on a generalized linear model [41]. We found 1,012 exons spread across 685 genes were differentially used between infected and cured lines, though genes containing differentially used exons were not significantly enriched for any GO terms.

To examine if there was a network-level change associated with *Wolbachia* infection status, we performed a weighted gene co-expression network analysis (WGCNA) [42] using both cured and infected expression data. This method constructs clusters of correlated genes based on their expression levels and relates these gene clusters, or modules, to external traits [42], such as infection status in this study. We identified a total of 28 modules, in which one module containing 481 genes was significantly enriched for expression changes according to infection status (*r* = 0.96, *P* = 0.002). We found no enriched GO terms in this module, though this may in part be due to 160 of the genes having no known annotations. This module includes 44 DEGs, which is a highly significant enrichment (Fisher’s exact test, Odds ratio = 75.84, *P* < 10^−15^). The majority of genes in this module are up-regulated in the infected lines (336 out of 481 total genes and 35 out of 44 DEGs, *P* < 10^−15^ and 9.82x10^-8^ respectively, χ^2^ test). This module also included 2 DMGs, though this overlap was not statistically significant (Fisher’s exact test, Odds ratio = 0.64, *P* = 0.82).

### Differential methylation significantly associates with differential exon usage but not differential gene expression

Next, we investigated relationships between expression and methylation that were correlated with *Wolbachia* infection. In both the cured and infected lines, there is a clear separation of methylated and unmethylated genes, with methylated genes tending to be more highly expressed than unmethylated genes, regardless of *Wolbachia* infection status (Fig 2C; S3 Fig). This is consistent with observations in other hymenopterans [43–45] and in the majority of invertebrates investigated so far, where methylated genes are more highly and constitutively expressed [46, 47].

We then asked if differentially methylated genes were also differentially expressed, as we might expect changes in DNA methylation to directly modulate gene expression. In contrast, we found that DEGs and DMGs were completely distinct and there was no overlap between them (Fig 2C; S4 Fig). While none of the DEGs were significantly differentially methylated, 32 of the 39 up-regulated DEGs (82%) in the infected lines also had higher (but non-significant) levels of fractional methylation compared to the cured lines. This constitutes a significant bias towards higher levels of methylation in infected lines (χ^2^ test, *P* = 5.48x10^-8^), demonstrating consistency between the epigenomic and transcriptomic changes associated with *Wolbachia* infection.

**Fig 3:**
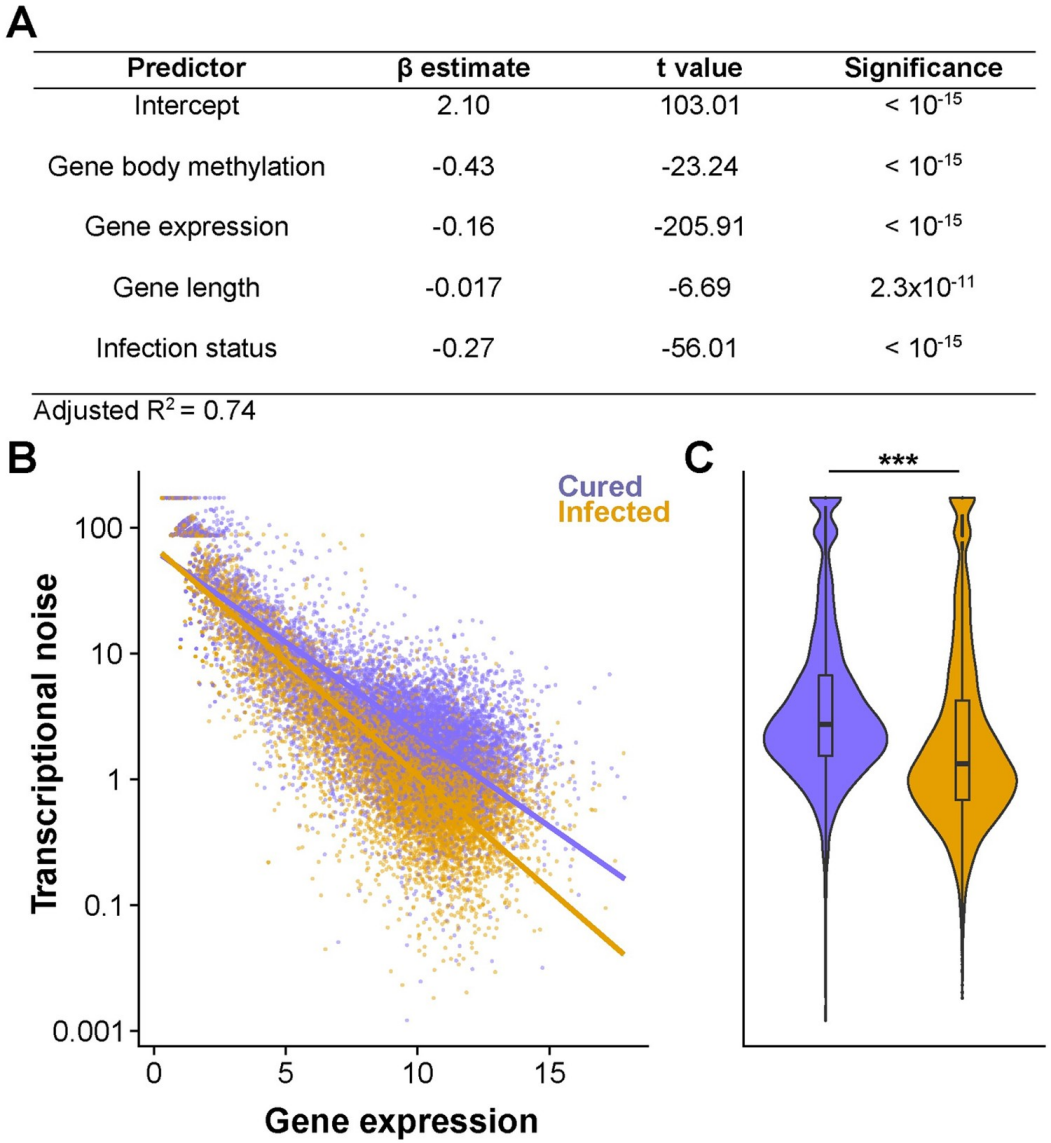
Transcriptional noise is negatively correlated with gene body methylation, gene expression, gene length, and *Wolbachia* infection. A) Summary of multiple linear regression modeling transcriptional noise, represented as the percent coefficient of variation of gene expression. B) Transcriptional noise is lower in infected lines and decreases more sharply with increased gene expression (Regression coefficients for cured and infected lines are -0.83 (*P* < 10^−15^) and -0.88 (*P* < 10^−15^), respectively). Transcriptional noise and gene expression are log_10_ and log_2_ transformed to improve clarity (see Methods). C) Violin plot with an imbedded boxplot demonstrating that transcriptional noise in infected lines is lower than that in cured lines (Student’s t-test, *P* < 10^−15^).

We also observed an intriguing pattern with regard to the variability in gene expression, or “transcriptional noise”. Previous studies have proposed that DNA methylation of genic regions, which is often referred to as ‘gene body methylation’, might reduce transcriptional noise [48, 49]. It is notable that we observed hypermethylation of CpGs associated with *Wolbachia* infection (see above). We thus tested whether transcriptional noise is correlated with *Wolbachia* infection, by constructing a linear model using transcriptional noise (measured by the coefficient of variation of expression [49]) as the response variable. We included gene body methylation (measured as mean DNA methylation levels of CpGs within each gene), gene expression, gene length, and infection status as explanatory variables (Methods). The results show that each explanatory variable was highly significant, indicating that transcriptional noise is negatively correlated with gene body methylation, gene expression, gene length, as well as *Wolbachia* infection (Fig 3A). We show, for the first time, that *Wolbachia* infection is accompanied by a decrease in transcriptional noise (Fig 3B and 3C), even after accounting for the effect of increased DNA methylation.

**Fig 4:**
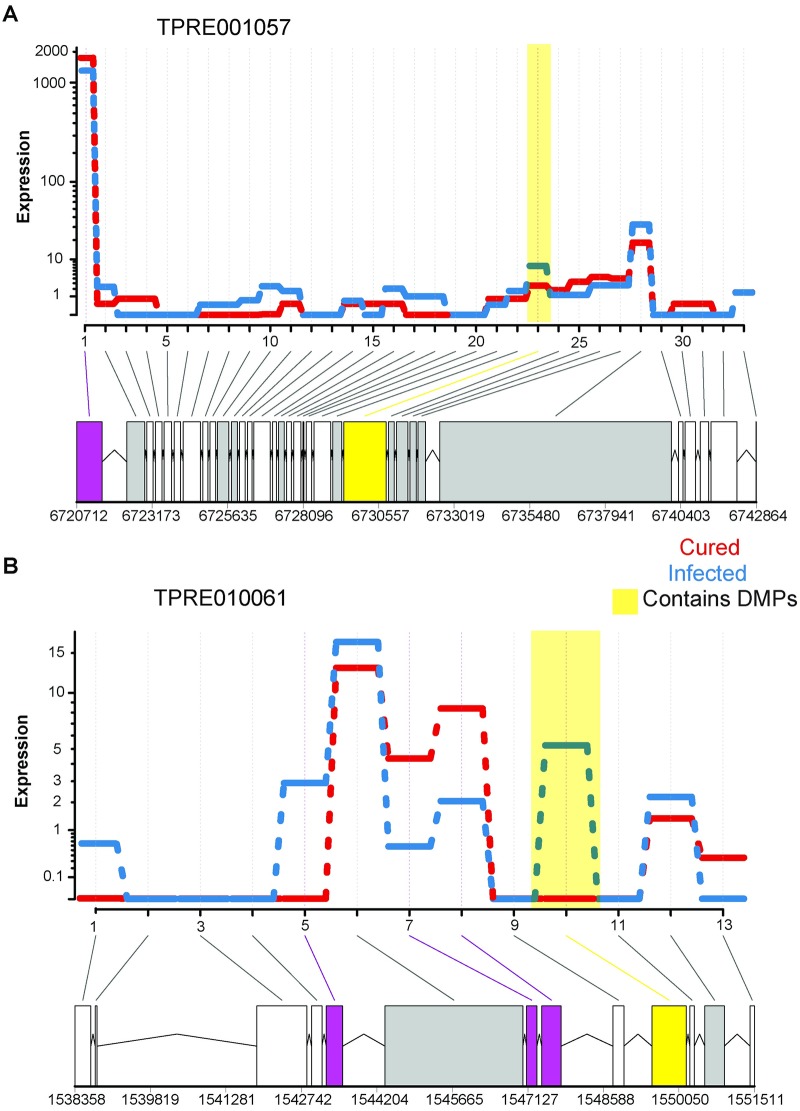
Representative genes showing differential exon usage between cured and infected *Trichogramma*. Boxes filled in purple are differentially used exons. Among the differentially used exons, those that contain DMPs are shown in yellow-filled boxes. A) Gene TPRE001057, ortholog of *D*. *melanogaster CG14299*, contains 2 differentially used exons, exons 1 and 23 (exon numbers are shown as integers on the x-axis). The exon 23 contains 6 DMPs is highlighted with a yellow-filled box. B) Gene TPRE010061, ortholog of *D*. *melanogaster Mzt1*, contains 4 differentially used exons (exons 5, 7, 8, and 10). The exon 10 has 8 DMPs and shown in yellow-filled box.

Another known impact of DNA methylation is differential exon usage [50–53], which could occur due to changes in splicing, or changes in expression levels of specific splice variants. Additionally, three of the genes with the highest numbers of DMPs have predicted functions related to the regulation of splicing (S6 Table). Of the 1,012 exons spread across 685 genes that were significantly differentially expressed between infected and cured lines, only 5 exons contained DMPs. However, given the small number of overall DMPs (340 out of the total 106,475 CpGs analyzed), this represents a statistically significant overlap (Odds ratio = 4.40, Fisher’s exact test, *P* = 0.0071). Since DNA methylation within one exon can affect splicing of other exons in the same gene [54–56], we further examined whether genes harboring differentially used exons also include differentially methylated positions, regardless of whether it was the differentially used exon that directly contained the DMP(s). In other words, we examined the overlap between DMGs and genes with evidence of differential exon usage. Indeed, this overlap was significant (14 genes, Fisher’s exact test, Odds ratio = 3.29, *P* = 3.14 x 10^−4^). In comparison, only 3 genes with differentially used exons were DEGs (Odds ratio = 0.87, Fisher’s exact test, *P* = 0.67). Two examples of the relationship between DMPs and differential exon usage are depicted in Fig 4. Gene *TPRE001057*, whose *D*. *melanogaster* ortholog is involved in autophagy (CG14299), harbors 2 differentially used exons, and one of them (exon 23) harbors 6 DMPs (Fig 4A). Another gene *TPRE001061*, an ortholog of *D*. *melanogaster Mzt1* involved in male mating behavior, has a total of 4 differentially use exons, where exon 10 contains 8 DMPs (Fig 4B). Interestingly, for both genes, the exons that contain DMPs had higher expression in the infected lines, which is consistent with the higher level of fractional methylation of DMPs in the infected lines. Overall, 614 of the 1,012 (60.1%) differentially used exons had increased usage in the infected lines (χ^2^ test, *P* = 10^−15^).

## Discussion

Symbiotic microbes are increasingly appreciated as driving forces in evolution [57]. *Wolbachia*’s widespread presence across arthropods and nematodes, and its primarily maternal transmission, make it a particularly interesting model to study the mechanisms behind infection and host manipulation. The induction of parthenogenesis has been described across several orders of arthropods [3], but is poorly understood, with the lack of traditional insect model organisms infected by parthenogenesis-inducing *Wolbachia* being a major challenge. Here, we developed an introgression scheme to delineate the global methylome and transcriptome modifications that follow a drastic transition from sexual to asexual reproduction due to the association with *Wolbachia*. A unique advantage of our introgression scheme and choice of organism is that we can examine molecular changes free from genetic background effects [25, 26] and in an organism with moderate amounts of genic DNA methylation [27].

Using this approach, we demonstrate clear changes of epigenomes and transcriptomes. Differentially expressed genes following *Wolbachia* infection tended to be unmethylated and are enriched for those with unique evolutionary patterns in the *Trichogramma* lineage [27], pointing to a lineage-specific response to *Wolbachia* infection in this group of insects. Previous studies have determined that the *Wolbachia* strains infecting *Trichogramma* wasps form a unique monophyletic clade within the *Wolbachia* phylogeny [58]. This pattern is relatively unusual, as *Wolbachia* undergoes host-switching at a relatively high rate for an intracellular, maternally transmitted reproductive symbiont [58–61]. If these *Wolbachia* strains have mechanisms for facilitating infection that specifically target processes unique to *Trichogramma*, it would explain why there have been no horizontal transfers back out of *Trichogramma* hosts. Given that there are limited functional predictions available for these lineage specific genes, reverse genetics approaches will be particularly powerful for elucidating their role in the maintenance of and/or the response to *Wolbachia* infection and the induction of parthenogenesis.

We found 84 genes that were differentially methylated between infected and cured *Trichogramma* wasps. This number is on par with that associated with *Wolbachia* infection in *Aedes* mosquitoes (63 genes in *A*. *aegypti* [20]) and that associated with viral infection in honey bee [62], indicating that approximately 1–2% of analyzed genes change DNA methylation in response to pathogenic infection in these species. Interestingly, the number of DNA methylation changes associated with human diseases tended to be in much smaller proportions (less than 0.5%, e.g., [63–65]). In contrast to differentially expressed genes, differentially methylated genes are evolutionarily conserved genes and included those involved in processes such as oogenesis, meiosis, and cell division, which is consistent with the proposed role of *Wolbachia* in inducing parthenogenesis via disruption of chromosome segregation during oocyte mitotic divisions [15]. The list of differentially methylated genes includes those involved in core eukaryotic processes, and thus potentially could affect both host chromosome segregation and transmission of *Wolbachia* to daughter cells.

While we show there is no direct correspondence between differential DNA methylation and differential gene expression, our study offers novel insight into how these two processes might be linked. We show that *Wolbachia* infection increases DNA methylation in *Trichogramma*. *Wolbachia* infection was similarly associated with increase in DNA methylation in *Drosophila* testis [37] and virus-infected honey bees [62]. In addition, *Wolbachia* infection is significantly negatively associated with transcriptional noise, suggesting that the presence of *Wolbachia* reduces the variability in gene expression, after accounting for the overall increase in methylation. However, it is also possible that the observed methylation changes may be a result of changes in physiology associated with *Wolbachia* infection and not necessarily the cause of any *Wolbachia*-mediated changes to the host. Though further experiments with larger numbers of samples will be necessary to validate and explain this observation, it provides another possible avenue of research for exploring the wide-ranging effects of *Wolbachia* infection. Moreover, we show that exons that are differentially used according to *Wolbachia*-infection status often occurred in the same genes that contained differentially methylated cytosines. Even though our current data are insufficient to explicitly identify alternatively spliced transcripts (due to the lack of detailed annotations), these results indicate that differential DNA methylation may regulate the isoforms of genes that are used, thus favoring different splice variants at certain loci. Our results provide a direct support to a link between differential DNA methylation and differential exon usage in insects [51, 62, 66], by controlling for genetic background effects. However, we note that only a small portion of differentially methylated positions appear to be associated with the differential usage of exons. Additionally, it is of note that our data were generated by pooling of individuals, which was necessary given the minute size of *Trichogramma*. While each biological replicate was clonal and thus the genomes are identical baring any mutations, there may be heterogeneity in the methylation signature (perhaps due to fluctuations in *Wolbachia* titer) that masks some links between methylation patterns and transcriptomic signatures, such as exon usage. Additional studies using more targeted approaches with single individuals or specific tissues will be useful for resolving these links.

Another unique feature of our experimental set-up is the use of introgression to generate genetically identical lines with and without *Wolbachia*. While this was required here (as the original *Wolbachia*-infected lines could not be cured of *Wolbachia* due to mutations in the wasp genome), there are likely distinct signatures of the infection in these introgressed lines (that now have *Wolbachia* associated with a different genomic background) as compared to the naturally infected lines. While the introgression was controlled for (i.e., both infected and cured lines had the same combination of nuclear and mitochondrial genomes), the signatures of infection in the “new” association may be different than *Wolbachia*-host associations that are long co-adapted. Comparing “new” vs co-adapted *Wolbachia* associations may be a useful tool in the future to identify how *Wolbachia* and host evolve after the onset of a new infection. There are many previous studies that identify genotype-by-genotype effects in *Wolbachia*-host associations (for a review, see [67]), so identifying commonalities and differences between associations will be useful for understanding how *Wolbachia* manipulates diverse hosts.

Our results will direct mechanistic investigations focused on the induction of symbiont-mediated parthenogenesis, and further our understanding of the relationship between epigenetics and gene expression in arthropods. While there are not currently any genetic tools for *Trichogramma* or *Wolbachia*, there is the potential to use reverse genetics (e.g., RNAi) in *Trichogramma*, or surrogate systems (e.g., *Nasonia*, *Drosophila*, yeast). Knockdown of target gene expression in *Trichogramma* will be useful in interrogating the contribution of particular proteins to *Wolbachia* establishment and reproductive manipulation. Expression of *Wolbachia* genes in surrogate systems is also a powerful tool for identifying bacterial proteins that may interface with host biology [10, 68].

## Materials and methods

### *Trichogramma* lines and rearing

*Trichogramma pretiosum* colonies are maintained in 12 x 75 mm glass culture tubes plugged with cotton, and incubated at 24°C with a light:dark cycle of 16:8 hours. Every 11 days, colonies are given fresh honey and egg cards to parasitize made of UV irradiated *Ephestia kuehniella* eggs (Beneficial Insectary, Guelph, Canada) adhered to card stock by double-sided tape. Four different isofemale lines were used in experiments (Insectary, ES865, CA9, and CA29). The “Insectary” line was initiated by a single female collected from the Puira Valley of Peru, and has been kept in an insectary since 1966 [15]. The “ES865” line was initiated by a single female collected in Oahu, Hawaii, USA in 2011 that emerged from a Lycaenid egg on *Crotalaria assamica*. Both the Insectary and ES865 lines are infected with parthenogenesis-inducing *Wolbachia*, as witnessed by the production of female offspring from virgin mothers and confirmed molecularly by PCR [69]. Additionally, all attempts at establishing sexually reproducing lines (using rifampicin) have failed (as seen in [70]). Males are produced, but self-sustaining, sexually reproducing lines have not been possible. The “CA9” line and the “CA29” are both highly inbred, sexually reproducing lines that each originated from a different sibling-mated female collected in Irvine, California, USA in 2008. The females emerged from *Manduca sexta* eggs collected from tomato plants. These lines are not infected with *Wolbachia*: virgin females produce only male offspring. Species identification and *Wolbachia* infection status were confirmed using molecular methods, as described previously [28, 71–73].

### Introgression of *Wolbachia* into sexual backgrounds

We introgressed the sexual CA9 nuclear genome into the asexual ES865 cytoplasm, and the sexual CA29 nuclear genome into the asexual Insectary cytoplasm (Fig 1A). These pairs were chosen based on the ability to differentiate them at the A9 microsatellite locus, a size-diagnostic amplicon routinely used for identifying unique isofemale lines of this wasp [71]. For example, the Insectary line has an A9 microsatellite of 312 bp in length, and the CA29 line has an A9 microsatellite of 220 bp in length–allowing us verify fertilization during introgression: a critical step because *Wolbachia* induces diploidization of unfertilized eggs, and thus female offspring are produced from both fertilized and unfertilized eggs [16]. To perform the introgression, virgin females from the “cytoplasm-donating line” were mated to virgin males of the “nuclear-genome-donating line”. This cross resulted in female offspring that are either heterozygous (due to fertilization), or homozygous (due to *Wolbachia*-mediated gamete duplication). These females, with undetermined zygosity, were isolated as virgins and allowed to parasitize egg cards for 24 hours, allowing us to determine how efficiently *Wolbachia* induces parthenogenesis (as inferred by sex ratio of the resulting offspring). After 24 hours of reproduction as virgins, wasps were mated to virgin males from the appropriate “nuclear-genome-donating line” (either CA9 or CA29), and then allowed to parasitize a separate egg card for the next 24 hours. After these two reproductive periods, the mated females were removed from their egg card, and used for DNA extraction and A9 microsatellite PCR assays, as described previously [71]. At each step of the introgression, we identified mothers who were heterozygous (indicating she was produced via fertilization), and then isolated virgin females originating from her “mated” egg card. These females, again with undetermined zygosity, were handled in the same manner as her mother: 24 hours on an egg card as a virgin to check sex ratio and fecundity, followed by mating and 24 hours on a fresh egg card to continue the introgression (Fig 1B).

After seven generations of introgression, we initiated three introgressed isofemale lines, each derived from a parallel, independent introgression, and grew up the lines over a period of three generations, following standard rearing protocols detailed above. After three generations, the lines were split, and one replicate was cured with rifiampicin, as described previously [4]. The antibiotic treatment protocol takes three generations to complete. We verified successful curing of the *Wolbachia* infection using protocols described above. Finally, after antibiotic treatment was complete, we allowed wasps to recover from the antibiotics for three generations prior to sequencing.

Sex ratio and fecundity were tracked over the course of the introgression to monitor the level of parthenogenesis induced by *Wolbachia* (sex ratio), and any potential cyto-nuclear incompatibilities caused by interactions between the cytoplasm (mitochondria and *Wolbachia*) and the nucleus (fecundity, as measured by the number of offspring that emerged as adults). Sex ratio and fecundity measurements were taken from virgin wasps after exposure to an egg card for 24 hours, as described above. Variation in fecundity or sex ratio was assessed with a generalized linear model, including “generation” as a fixed effect, and using either a Gaussian or binomial error distribution, respectively. Separate GLMs were run for the ES865 and Insectary datasets. Analyses were carried out in R version 3.5.0 [74], using the ‘stats’ package. The introgressed lines from the ES865 X CA9 wasps had very low fecundity, and we were unable to expand the colonies sufficiently for further experimentation. Thus, the CA29 X Insectary introgressed lines were chosen for further experimentation.

### Nucleotide extractions

To assess signatures of genome-wide cytosine methylation and gene expression, we extracted DNA and RNA from pools of *T*. *pretiosum* females. Newly emerged wasps (less than 48 hours post emergence and having had no exposure to a fresh egg card) were collected in a fresh culture vial and placed on a cooling pad for sex sorting, as determined by antennal morphology. Only females were found in the *Wolbachia*-infected lines, while the cured lines contained both males and females. Approximately 500 females (each female is ~0.3 mm long [75]) were collected for each biological replicate (total of six: three infected, three cured), and flash frozen in liquid nitrogen. That pool was homogenized, then split evenly between DNA and RNA extractions with Qiagen DNeasy and RNeasy kits, respectively, according to manufacturer’s instructions, and including the appropriate on-column RNase or DNase digestion to remove RNA and DNA contamination. The final number of extractions, each of which was made into a library (see below), was 12: DNA and RNA samples for three biological replicates each of infected and cured lines.

### Transcriptome sequencing

Strand specific RNA-Seq libraries were prepared by NovoGene (https://en.novogene.com/, Chula Vista, CA) using a standard eukaryotic preparation workflow. Briefly, mRNA was enriched for using oligo(dT) beads, and then fragmented randomly by adding fragmentation buffer. cDNA was synthesized by using mRNA template and random hexamers, after which a custom second-strand synthesis buffer (Illumina), dNTPs, RNase H and DNA polymerase I were added to initiate second-strand synthesis. After a series of terminal repair, A ligation and sequencing adaptor ligation, the double-stranded cDNA library was completed through size selection and PCR enrichment. The final library quality and quantity was assessed using an Agilent 2100 Bioanalyzer, and Qubit 2.0, respectively [76, 77]. Libraries were multiplexed and sequenced on an Illumina HiSeq with paired-end 150 base pair reads. This produced between 30–42 million paired-end reads per sample.

### Whole-genome sequencing

Genomic libraries were made following a modified version of an Illumina sequencer compatible protocol [78]. The extracted DNA was fragmented by S-series focused ultrasonicator (Covaris) using the 200bp-target peak size protocol. Fragmented DNA was then size selected (200bp-600bp) with an Agencourt AMPure XP bead-based (Beckman Coulter Cat. No. A63880) size selection protocol [78]. The DNA End repair step was performed with End-It DNA end repair kit (Epicentre, Cat. No. ER81050. After the end repair step, A-tailing (NEB, cat. No. M0202) and ligation steps were performed to ligate methylated adaptors.

### Whole genome bisulfite sequencing

We followed a previously published protocol [78] to perform whole genome bisulfite sequencing (WGBS). Briefly, bisulfite treatment of genomic DNA was performed using the MethylCode Bisulfite Conversion Kit (Life technologies). Purified genomic DNA was treated with CT conversion reagent in a thermocycler for 10 minutes at 98°C, followed by 2.5 hours at 64°C. As a control for bisulfite conversion, 10ng of unmethylated lambda phage DNA (Promega, Cat. No. D1501) was added to the 1μg of input DNA. Bisulfite-treated DNA fragments remain single-stranded as they are no longer complementary. Low-cycle (4–8) PCR amplification was performed with Kapa HiFi Uracil Hotstart polymerase enzyme (KAPA Biosystems, cat. No. KK2801) which can tolerate Uracil residues. The final library fragments contain thymines and cytosines in place of the original unmethylated cytosine and methylated cytosines, respectively. The methylome libraries were diluted and loaded onto Illumina HiSeq X system for sequencing using 150bp paired-end reads. We received 100–200 million paired-end reads per sample. Bisulfite conversion efficiencies are all greater than 99.8% (S3 Table).

### Creating alternative reference genome

We used the GATK best practices pipeline [79] to discover SNPs in the CA29 line and incorporated them into the original *Trichogramma* genome (the Insectary line) [27] to create an alternate reference genome for subsequent RNA-Seq and WGBS alignment. Briefly, CA29 reads were aligned to the Tpre_1.0 reference genome using BWA-MEM with option–M enabled. Duplicate reads were marked with Picard tools and INDELs were realigned to eliminate mapping artifacts with GATK. Base recalibration was performed with 3 rounds of bootstrapping with the resulting variants used as substitutes for the gold standard variants in the HaplotypeCaller SNP calling algorithm. The final lists of SNPs were substituted into the original Tpre_1.0 reference using GATK’s built-in FastaAlternateReferenceMaker tool.

### Transcriptomic data analysis

Reads were cleaned of adaptor sequence and quality filtered with Trimmomatic v.0.35 [80], using a sliding window of 4 bases and cutting when the average quality dropped below 20, employing a minimum read length of 40 bases. Cleaned reads were mapped to the CA29 alternate *T*. *pretiosum* reference genome (see above) [27] with tophat2 v. 2.2.1 [81], and mappings to coding regions were quantified with HTSeq’s ‘count’ feature [82]. Differential expression was assessed with DESeq2 [40], including infection status (either *Wolbachia* positive or *Wolbachia* negative) as a factor. DESeq2 and graphics were run in R version 3.3.2 [74]. Gene expression was measured by normalizing counts based on library sizes using the “estimateSizeFactors” function from the DESeq2 [40] package.

To assess expression differences at the exon level, we utilized the DEXseq [41] package. Differences in the level of gene expression between DEGs, DMGs, and genes that fell into neither of those categories were assessed with a one-way ANOVA using log-transformed gene expression values, and Tukey’s post hoc tests. Exon-level reads were counted using the alignments from our RNA-seq analysis and normalized using the “estimateSizeFactors” function from DESeq2 [40]. Exon usage was modeled with the following generalized linear model: ~ sample + exon + infection status:exon. Exonic regions were considered to be significant at a FDR-adjusted [83] level of 0.05.

### Transcriptional noise analysis

Transcriptional noise was measured as the percent coefficient of variation of gene expression [49] and used as the response variable in the following multiple linear regression model: log_10_(transcriptional noise) ~ gene body methylation + log_2_(gene expression) + log_10_(gene length) + *Wolbachia* infection status. Gene body fractional methylation was calculated as the mean fractional methylation of all CpGs within each gene [49] and gene expression was measured as described above. Analysis was performed in R version 3.3.2 [74] using the default ‘lm’ function.

### WGBS data processing

Raw reads were assessed for quality using FastQC and trimmed and filtered for quality and adapter sequences using Trim Galore! [84]. Bismark was then used to align reads to the CA29 alternate *T*. *pretiosum* reference genome (see above) with parameters—score_min L,0,-0.4 [85]. Additionally, we aligned our reads to the unmethylated lambda genome (GenBank Accession: J02459.1) to estimate the bisulfite conversion efficiency. Alignments were deduplicated and C and T counts for CpGs on plus and minus strands were combined using custom scripts (https://github.com/soojinyilab/miscellaneous-scripts/blob/master/combine_CpGs.pl) and classified as either “Methylated” or “Unmethylated” using Bis-Class for downstream analyses [30]. Detailed information regarding read numbers, alignment efficiency, bisulfite conversion rates and other methylation statistics can be found in S3 Table.

### Genomic mapping of introgression from bisulfite sequencing data

In order to identify paternal (introgressed) and maternal (non-introgressed) genomic regions present in each of our introgressed isofemale lines, we mapped WGBS reads of each sample to both the maternal and paternal genomes separately. We then used BS-SNPer [29], a tool that can identify polymorphisms from bisulfite-sequencing data, on each maternal and paternal alignment and used stringent parameters to filter for high confidence SNPs. We required putative SNPs to be homozygous, have at least 10X sequencing depth, and a sequencing quality score of at least 30 to pass the filtering step. We determined the allelic origin of each SNP by comparing the base call to the original SNP list generated between maternal and paternal genomes (see Creating alternative reference genome section above). Maternal SNPs occurred in clusters, as evidenced by the short distance between adjacent SNPs (S1 Table and S4 Fig). We identified putatively non-introgressed regions as those starting from a maternal SNP, and added the next maternal SNP within a certain threshold distance of the previous maternal SNP until a paternal SNP was reached. We removed all regions putatively non-introgressed using a 10kb threshold from all analyses.

### Gene ontology analyses

Gene Ontology (GO) terms were mapped to the coding sequences using Blast2GO [86] after blastp searches against the NCBI non-redundant database, and interpro scans with the Blast2GO software, the same as done previously for *T*. *pretiosum* [27]. BiNGO [87] was used to identify significantly overrepresented GO terms in sets of differentially expressed or differentially methylated genes, using all genes in the genome as the background. Testing was performed with hypergeometric tests and Benjamini & Hochberg FDR correction, at a corrected significance level of 0.05 [83]. In addition to using GO terms to classify genes, the previously published orthology data was used to determine whether genes were specific to the *Trichogramma* lineage [27]. *Trichogramma*-specific genes are as previously described, and include those that are: A) truly unique to the *Trichogramma* lineage, B) genes that have orthologs in other hymenopteran taxa but evolved rapidly in *Trichogramma* such that orthology was difficult to detect, and/or C) genes that are members of gene families that expanded significantly in copy number in the *Trichogramma* lineage, resulting in one more *Trichogramma*-specific paralogs not present in other hymenopterans.

### Differential methylation analysis

To improve the statistical power to detect differential DNA methylation, we followed a suggested protocol [88]. First, we selected 106,475 CpG sites that were methylated according to Bis-Class [30] in at least one of the six samples. Next, we used a beta-binomial regression method to identify individual CpGs that were differentially methylated between infected and cured *Trichogramma* lines. We refer to these individual CpGs as ‘differentially methylated positions (DMPs)’. The RADMeth (logit link) package [89] was applied using default parameters and adjusted for multiple testing using the option -bins 1:200:200. This method utilizes a beta-binomial regression model and has been shown to excel at its ability to detect differential DNA methylation [89]. DMPs were corrected for multiple testing at an FDR-adjusted [83] level of 0.05, which roughly corresponds to *P* = 5 x 10^−5^.

### Weighted correlation network analysis (WGCNA)

To detect clusters of genes that are highly correlated in their expression, WGCNA was performed using library normalized counts assembled via the HTSeq [82] package from the transcriptome analysis step and using infection status as the sole external factor. Normalized counts were calculated by dividing the total count by the size factor obtained using the “estimateSizeFactors” function from the DESeq2 [40] package. Module detection was done using a soft thresholding power *β* of 16, a minimum module size of 20, and default parameters for the rest of the options (S5 Fig).

## Supporting information

S1 Fig Distribution of the number of DMPs per DMG. DMGs have on average 4.5 DMPs and up to 18 DMPs.
(PDF)

S2 Fig Volcano plot of all genes based on their log_2_ fold-change and adjusted P-values. Differentially expressed genes were classified at an adjusted P-value of < 0.05.
(PDF)

S3 Fig Methylated genes (>0.004 gene body methylation) exhibit higher gene expression than unmethylated genes (<0.004 gene body methylation) in both A) cured and B) infected wasps. Variability of gene expression is reduced in methylated genes when measuring gene body methylation by the number of methylated sites in C) cured and D) infected wasps.
(PDF)

S4 Fig Density plots of distances between adjacent maternal SNPs for samples in line A, both of which contain a large number of maternal SNPs. Samples are labeled using a 2-letter system–the first letter indicates the line (‘A’, ‘B’, and ‘C’) while the second letter indicates the infection status (‘i’ for infected and ‘c’ for cured). 89.5% of adjacent maternal SNPs are within 2kb of each other while 98.8% are within 10kb of each other.
(PDF)

S5 Fig Scale-free fit index and mean connectivity as a function of the soft-thresholding power β. β was chosen based on when the value of when R^2^ begins to flatten near a relatively high value (indicated by the red line at R^2^ = 0.80).
(PDF)

S1 Table Estimated number and size (in bp) of non-introgressed regions in each sample based on two threshold distances allowed between maternal SNPs (Materials and methods). Samples are labeled using a 2-letter system–the first letter indicates the line (‘A’, ‘B’, and ‘C’) while the second letter indicates the infection status (‘i’ for infected and ‘c’ for cured).
(PDF)

S2 Table Comparison of significant findings before and after removing genes and mCGs from putative non-introgressed regions in line A (10kb threshold; see S3 Table). (PDF)

S3 Table Summary statistics of BS-seq alignment and basic CpG coverage and methylation information. Samples are labeled using a 2-letter system–the first letter indicates the line (‘A’, ‘B’, and ‘C’) while the second letter indicates the infection status (‘i’ for infected and ‘c’ for cured).
(PDF)

S4 Table Detailed results of the various differential methylation, expression, and exon usage analyses as well as lists of gene names significant from each. (XLSX)

S5 Table Top ten significant biological processes GO terms for DMGs based on FDR values. (PDF)

S6 Table List of the top 10 genes ordered by the number of DMPs contained and their corresponding *Drosophila melanogaster* ortholog and functional annotation. (PDF)

S7 Table *Trichogramma* lineage-specific differentially expressed genes. (PDF)
