# Supplementary material for: Distinct epigenomic and transcriptomic modifications associated with Wolbachia-mediated asexuality
Source: PLoS Pathog. 2020 Mar 18;16(3):e1008397. doi: 10.1371/journal.ppat.1008397 (PMC7105135; doi:10.1371/journal.ppat.1008397)

Supplemental Figure 1. Distribution of the number of DMPs per DMG. DMGs have on average 4.5 DMPs and up to 18 DMPs.

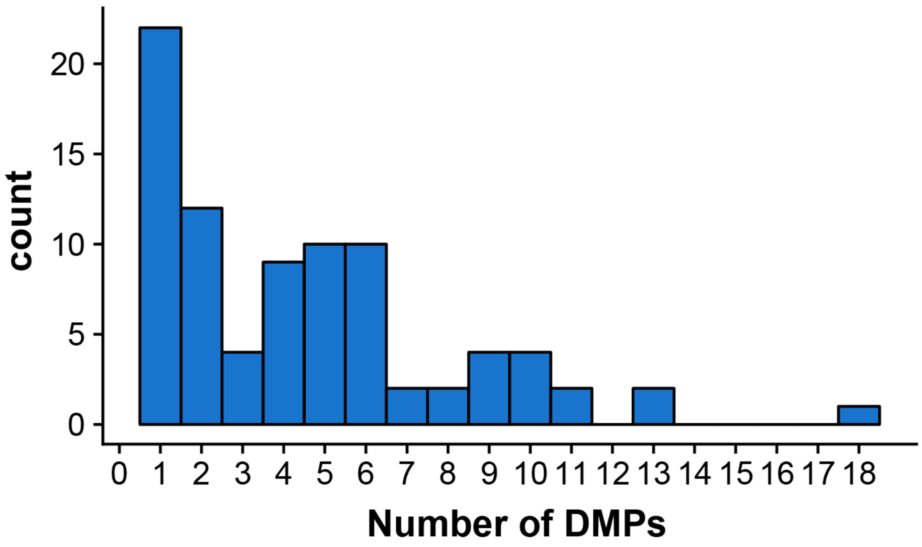

Supplement: S1 Fig — DMGs have on average 4.5 DMPs and up to 18 DMPs. (PDF) [file ppat.1008397.s001.pdf]
