# Supplementary material for: Distinct epigenomic and transcriptomic modifications associated with Wolbachia-mediated asexuality
Source: PLoS Pathog. 2020 Mar 18;16(3):e1008397. doi: 10.1371/journal.ppat.1008397 (PMC7105135; doi:10.1371/journal.ppat.1008397)

Supplemental Figure 2. Volcano plot of all genes based on their $\log_2$ fold-change and adjusted P-values. Differentially expressed genes were classified at an adjusted P-value of < 0.05.

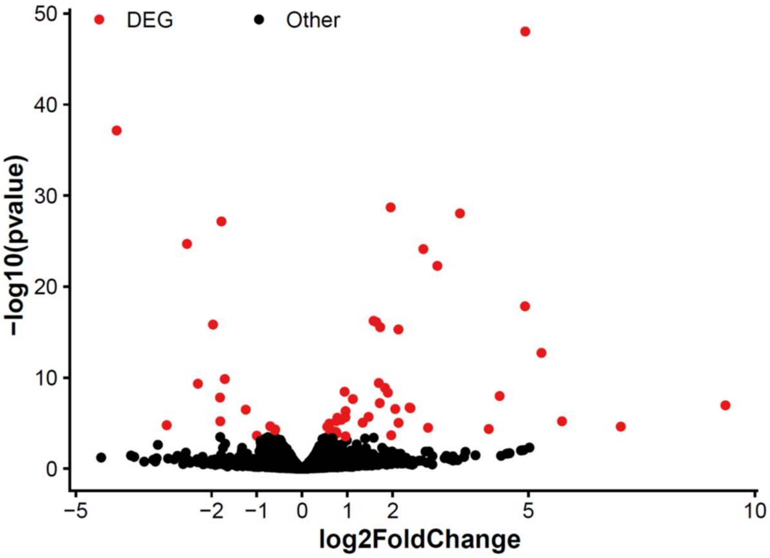

Supplement: S2 Fig — Differentially expressed genes were classified at an adjusted P-value of < 0.05. (PDF) [file ppat.1008397.s002.pdf]
