# Supplementary material for: Distinct epigenomic and transcriptomic modifications associated with Wolbachia-mediated asexuality
Source: PLoS Pathog. 2020 Mar 18;16(3):e1008397. doi: 10.1371/journal.ppat.1008397 (PMC7105135; doi:10.1371/journal.ppat.1008397)

Supplemental Figure 2. Methylated genes (>0.004 gene body methylation) exhibit higher gene expression than unmethylated genes (<0.004 gene body methylation) in both A) cured and B) infected wasps. Variability of gene expression is reduced in methylated genes when measuring gene body methylation by the number of methylated sites in C) cured and D) infected wasps.

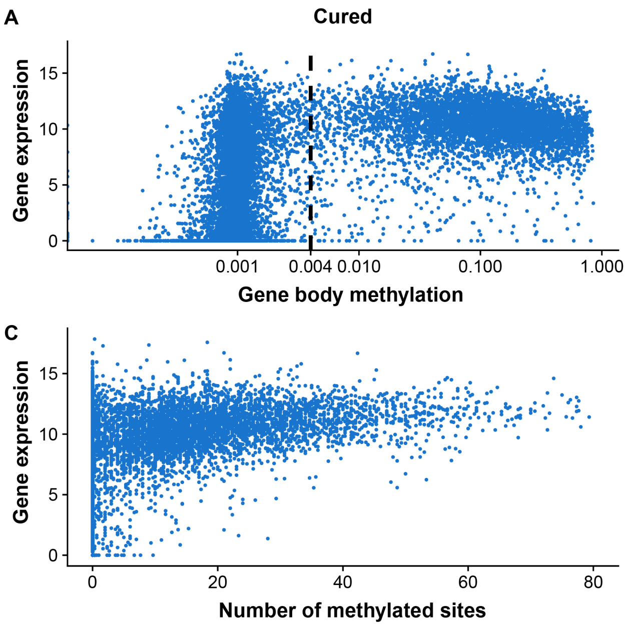

Supplement: S3 Fig — Methylated genes (>0.004 gene body methylation) exhibit higher gene expression than unmethylated genes (<0.004 gene body methylation) in both A) cured and B) infected wasps. Variability of gene expression is reduced in methylated genes when measuring gene body methylation by the number of methylated sites in C) cured and D) infected wasps. (PDF) [file ppat.1008397.s003.pdf]
