# Supplementary material for: Distinct epigenomic and transcriptomic modifications associated with Wolbachia-mediated asexuality
Source: PLoS Pathog. 2020 Mar 18;16(3):e1008397. doi: 10.1371/journal.ppat.1008397 (PMC7105135; doi:10.1371/journal.ppat.1008397)

Supplemental Figure 3. Density plots of distances between adjacent maternal SNPs for samples in line A, both of which contain a large number of maternal SNPs. Samples are labeled using a 2-letter system – the first letter indicates the line ('A', 'B', and 'C') while the second letter indicates the infection status ('i' for infected and 'c' for cured). 89.5% of adjacent maternal SNPs are within 2kb of each other while 98.8% are within 10kb of each other.

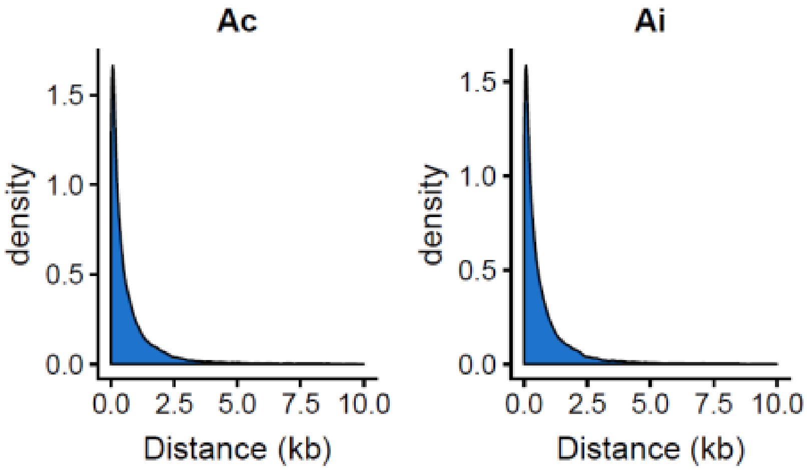

Supplement: S4 Fig — Samples are labeled using a 2-letter system–the first letter indicates the line (‘A’, ‘B’, and ‘C’) while the second letter indicates the infection status (‘i’ for infected and ‘c’ for cured). 89.5% of adjacent maternal SNPs are within 2kb of each other while 98.8% are within 10kb of each other. (PDF) [file ppat.1008397.s004.pdf]
