# Supplementary material for: Distinct epigenomic and transcriptomic modifications associated with Wolbachia-mediated asexuality
Source: PLoS Pathog. 2020 Mar 18;16(3):e1008397. doi: 10.1371/journal.ppat.1008397 (PMC7105135; doi:10.1371/journal.ppat.1008397)

Supplemental Figure 4. Scale-free fit index and mean connectivity as a function of the soft-thresholding power β. β was chosen based on when the value of when R^2 begins to flatten near a relatively high value (indicated by the red line at R^2 = 0.80).

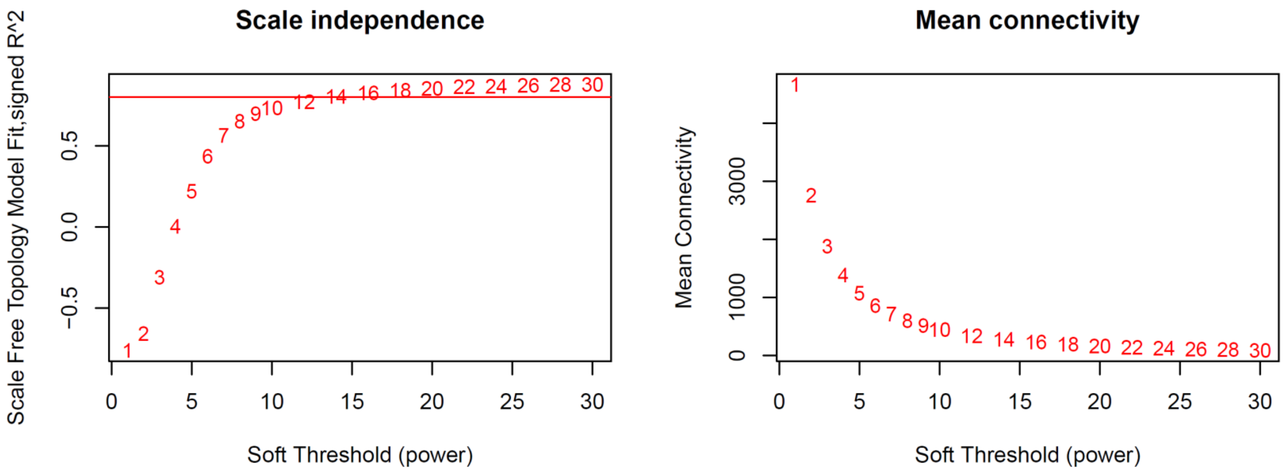

Supplement: S5 Fig — β was chosen based on when the value of when R2 begins to flatten near a relatively high value (indicated by the red line at R2 = 0.80). (PDF) [file ppat.1008397.s005.pdf]
