# Supplementary material for: Distinct epigenomic and transcriptomic modifications associated with Wolbachia-mediated asexuality
Source: PLoS Pathog. 2020 Mar 18;16(3):e1008397. doi: 10.1371/journal.ppat.1008397 (PMC7105135; doi:10.1371/journal.ppat.1008397)
Supplement: S1 Table — Samples are labeled using a 2-letter system–the first letter indicates the line (‘A’, ‘B’, and ‘C’) while the second letter indicates the infection status (‘i’ for infected and ‘c’ for cured). (PDF) [file ppat.1008397.s006.pdf]

Supplemental Table 1. Estimated number and size (in bp) of non-introgressed regions in each sample based on two threshold distances allowed between maternal SNPs (Materials and Methods). Samples are labeled using a 2-letter system – the first letter indicates the line ('A', 'B', and 'C') while the second letter indicates the infection status ('i' for infected and 'c' for cured).

				**2kb threshold**		**10kb threshold**	
**Sample**	**Number of maternal SNPs**	**Number of Paternal SNPs**	**Number of non-introgressed regions**	**Size of non-introgressed regions (bp)**	**% of non-introgressed genome**	**Size of non-introgressed regions (bp)**	**% of non-introgressed genome**
**Ac**	23,902	254,099	1,689	9,767,478	4.98	16,181,562	8.25
**Ai**	23,031	225,627	1,698	9,633,744	4.91	16,165,647	8.24
**Bc**	263	277,803	30	9,404	0.00479	20,818	0.0106
**Bi**	263	275,107	33	9,508	0.00485	25,602	0.0131
**Cc**	317	279,356	41	13,170	0.00671	15,467	0.00789
**Ci**	233	266,650	29	9,459	0.00482	20,871	0.0106
