# Supplementary material for: Distinct epigenomic and transcriptomic modifications associated with Wolbachia-mediated asexuality
Source: PLoS Pathog. 2020 Mar 18;16(3):e1008397. doi: 10.1371/journal.ppat.1008397 (PMC7105135; doi:10.1371/journal.ppat.1008397)
Supplement: S2 Table — (PDF) [file ppat.1008397.s007.pdf]

Supplemental Table 2. Comparison of significant findings before and after removing genes and mCGs from putative non-introgressed regions in line A (10kb threshold; see Supplemental table 3).

Analysis Method	Before removal	After removal	Overlap
**Differentially methylated positions**	306	340	306
**Differentially methylated genes**	74	84	74
**Differentially expressed genes**	57	59	49
**Differentially used exons**	1076 exons<br>742 genes	1012 exons<br>685 genes	1000 exons<br>678 genes
