# Supplementary material for: Distinct epigenomic and transcriptomic modifications associated with Wolbachia-mediated asexuality
Source: PLoS Pathog. 2020 Mar 18;16(3):e1008397. doi: 10.1371/journal.ppat.1008397 (PMC7105135; doi:10.1371/journal.ppat.1008397)
Supplement: S3 Table — Samples are labeled using a 2-letter system–the first letter indicates the line (‘A’, ‘B’, and ‘C’) while the second letter indicates the infection status (‘i’ for infected and ‘c’ for cured). (PDF) [file ppat.1008397.s008.pdf]

Supplemental Table 3. Summary statistics of BS-seq alignment and basic CpG coverage and methylation information. Samples are labeled using a 2-letter system – the first letter indicates the line ('A', 'B', and 'C') while the second letter indicates the infection status ('i' for infected and 'c' for cured).

Sample	Sequencing Library	Reads After Preprocessing	Duplication Level %	Alignment Efficiency %	Bisulfite Conversion Efficiency %	CpG Coverage[1]	Average CpG fractional methylation[1]	Methylated sites[1]
**Ac**	150 PE	102,095,911	R1 - 19.06<br>R2 - 9.62	83.9	99.90	80.69	0.00702	98,808
**Bc**	150 PE	113,371,000	R1 - 18.98<br>R2 - 16.82	81.9	99.87	93.92	0.0067	99,681
**Cc**	150 PE	120,857,668	R1 - 21.58<br>R2 - 18.69	81.8	99.91	100.35	0.00708	98,615
**Ai**	150 PE	65,015,257	R1 - 16.59<br>R2 - 10.40	81.7	99.89	47.9	0.00744	96,420
**Bi**	150 PE	112,865,282	R1 - 17.50<br>R2 - 14.99	80.4	99.88	86.67	0.00703	98,773
**Ci**	150 PE	84,926,702	R1 - 19.88<br>R2 - 16.65	81.3	99.88	67.56	0.00715	98,106
