# Supplementary material for: Distinct epigenomic and transcriptomic modifications associated with Wolbachia-mediated asexuality
Source: PLoS Pathog. 2020 Mar 18;16(3):e1008397. doi: 10.1371/journal.ppat.1008397 (PMC7105135; doi:10.1371/journal.ppat.1008397)
Supplement: S5 Table — (PDF) [file ppat.1008397.s010.pdf]

Supplemental Table 5. Top ten significant biological processes GO terms for DMGs based on FDR values.

GO accession	FDR-adjusted Q	Term
578	0.0379	Embryonic axis specification
9880	0.0379	Embryonic pattern specification
7316	0.0379	Pole plasm RNA localization
45451	0.0379	Pole plasm oskar mRNA localization
19094	0.0379	Pole plasm mRNA localization
9798	0.0379	Axis specification
7315	0.0389	Pole plasm assembly
7028	0.0389	Cytoplasm organization
7314	0.0434	Oocyte anterior/posterior axis specification
8358	0.0434	Maternal determination of anterior/posterior axis, embryo

[1]Numbers obtained following deduplication of reads and combining CpG read information from plus and minus strands
