# Supplementary material for: Distinct epigenomic and transcriptomic modifications associated with Wolbachia-mediated asexuality
Source: PLoS Pathog. 2020 Mar 18;16(3):e1008397. doi: 10.1371/journal.ppat.1008397 (PMC7105135; doi:10.1371/journal.ppat.1008397)
Supplement: S6 Table — (PDF) [file ppat.1008397.s011.pdf]

Supplemental Table 6. List of the top 10 genes ordered by the number of DMPs contained and their corresponding *Drosophila melanogaster* ortholog and functional annotation.

***T. pretiosum* ID**	***D. melanogaster* Symbol**	**DMPs**	**Function**	**Link**
**TPRE002596**	CG4896	18	Regulation of alternative splicing	https://www.uniprot.org/uniprot/Q8IPW6
**TPRE012191**	Chc	13	Clathrin coat assembly; compound eye development; Oocyte microtubule polarization; Sperm individualization;	https://www.uniprot.org/uniprot/P29742
**TPRE007614**	Rpii215	13	Subunit of RNA Polymerase II	https://www.uniprot.org/uniprot/P04052
**TPRE004555**	N/A	11	N/A	N/A
**TPRE001159**	Nmt	11	Adds myristoyl group to N-terminal of glycine; Dorsal closure;	https://www.uniprot.org/uniprot/O61613
**TPRE012609**	CG8258	10	Protein folding	https://www.uniprot.org/uniprot/Q7K3J0
**TPRE001893**	pAbp	10	Binds to poly(A) tail of mRNAs; mRNA splicing;	https://www.uniprot.org/uniprot/P11940
**TPRE001892**	CG11986	10	N/A	https://www.uniprot.org/uniprot/Q9VHI3
**TPRE001344**	CG7483	10	Chromatin organization; Exon-exon junction complex assembly; mRNA splicing; pole plasm oskar mRNA localization;	https://www.uniprot.org/uniprot/Q9VHS8
**TPRE011691**	ade5	9	'de novo' IMP biosynthetic process	https://www.uniprot.org/uniprot/Q9I7S8
