# Supplementary material for: Distinct epigenomic and transcriptomic modifications associated with Wolbachia-mediated asexuality
Source: PLoS Pathog. 2020 Mar 18;16(3):e1008397. doi: 10.1371/journal.ppat.1008397 (PMC7105135; doi:10.1371/journal.ppat.1008397)
Supplement: S7 Table — (PDF) [file ppat.1008397.s012.pdf]

Supplemental Table 7. *Trichogramma* lineage-specific differentially expressed genes.

Category	Genes
**“Rapidly evolving” Lineage Specific DEGs***	TPRE002398, TPRE002399, TPRE002442, TPRE002566, TPRE002879, TPRE003023, TPRE004094, TPRE004600, TPRE005031, TPRE005162, TPRE006114, TPRE006116, TPRE007067, TPRE007283, TPRE008446, TPRE008882, TPRE009686, TPRE009715, TPRE009787, TPRE010406, TPRE010451, TPRE010793, TPRE011077, TPRE011078
**DEGs truly unique to *Trichogramma****	TPRE000618, TPRE001270, TPRE002864, TPRE002880, TPRE003683, TPRE003720, TPRE004261, TPRE007488, TPRE008706
**DEGs that are members of gene families with unique paralogs in *Trichogramma****	TPRE008446, TPRE002442, TPRE010793, TPRE010347, TPRE005031, TPRE007067, TPRE009686, TPRE002399, TPRE002398, TPRE008882

*Designations according to Lindsey et al 2018, BMC Biology
